# Extracellular Vesicle Mediated Tumor-Stromal Crosstalk Within an Engineered Lung Cancer Model

**DOI:** 10.3389/fonc.2021.654922

**Published:** 2021-04-23

**Authors:** Kayla F. Goliwas, Hannah M. Ashraf, Anthony M. Wood, Yong Wang, Kenneth P. Hough, Sandeep Bodduluri, Mohammad Athar, Joel L. Berry, Selvarangan Ponnazhagan, Victor J. Thannickal, Jessy S. Deshane

**Affiliations:** ^1^ Department of Medicine, Division of Pulmonary, Allergy, and Critical Care Medicine, University of Alabama at Birmingham, Birmingham, AL, United States; ^2^ Department of Dermatology, University of Alabama at Birmingham, Birmingham, AL, United States; ^3^ Department of Biomedical Engineering, University of Alabama at Birmingham, Birmingham, AL, United States; ^4^ Department of Pathology, Division of Molecular and Cellular Pathology, University of Alabama at Birmingham, Birmingham, AL, United States

**Keywords:** tumor-stromal crosstalk, extracellular vesicles, 3D tissue models, lung carcinoma, tumor-immune regulation

## Abstract

Tumor-stromal interactions within the tumor microenvironment (TME) influence lung cancer progression and response to therapeutic interventions, yet traditional *in vitro* studies fail to replicate the complexity of these interactions. Herein, we developed three-dimensional (3D) lung tumor models that mimic the human TME and demonstrate tumor-stromal crosstalk mediated by extracellular vesicles (EVs). EVs released by tumor cells, independent of p53 status, and fibroblasts within the TME mediate immunomodulatory effects; specifically, monocyte/macrophage polarization to a tumor-promoting M2 phenotype within this 3D-TME. Additionally, immune checkpoint inhibition in a 3D model that included T cells showed an inhibition of tumor growth and reduced hypoxia within the TME. Thus, perfused 3D tumor models incorporating diverse cell types provide novel insights into EV-mediated tumor-immune interactions and immune-modulation for existing and emerging cancer therapies.

## Introduction

Lung cancer is the leading cause of cancer-related death in the United States (1). Non-small cell lung carcinoma (NSCLC), a heterogeneous group of lung malignancies, accounts for the majority of all diagnosed lung cancers (2, 3). Despite improvement in outcomes with targeted and combination therapies, the overall survival for NSCLC remains poor (4). Lung cancer progression and response to therapeutic intervention are influenced by tumor-stromal interactions within a tumor microenvironment (TME) consisting of tumor and stromal cell populations, within an extracellular matrix (ECM) scaffold (5, 6). Direct interaction between cancer and stromal cells, including cancer associated fibroblasts (CAFs) and immune cells, induces phenotypic changes in tumor cells, shaping metabolism, modulating invasion and proliferation, and regulating stemness to support tumor growth (7–12). Tumor-immune crosstalk regulates tumor progression, with cancer cells inducing phenotypic changes in resident and recruited immune cells to escape immune recognition. A better understanding of how immune populations shift in response to the TME and therapeutic intervention is critical for the development of new targeted therapies.

Extracellular vesicles (EVs) engage in indirect bidirectional cell-cell communication *via* transfer of bioactive molecules (13). Tumor cells coopt this mechanism of communication to influence immune modulation and activate fibroblasts. Tumor cell-derived EVs promote expansion of immunosuppressive cells, regulate macrophage polarization, and modulate antigen presentation to escape immunosurveillance (14–17). Additionally, EVs from other cells within the TME facilitate bidirectional crosstalk; EVs secreted by antigen-presenting cells assist in T cell priming, and those produced by fibroblasts facilitate metastasis (18, 19). Due to the complexity of the TME, additional mechanisms of EV-mediated tumor-stromal crosstalk remain undefined.

The complexity of solid tumors has limited many studies to two dimensional (2D) cell cultures that do not account for the three dimensional (3D) architecture or native tissue microenvironment (20). Further, the simplified microenvironment created by 2D culture impacts cellular behavior and does not mimic physical barriers within human tumors. To better model human lung tumors, we employed a tissue engineering approach to establish 3D culture models of NSCLC which include tumor cells, lung fibroblasts, and immune cells. We evaluate the impact of EV-mediated tumor-stromal crosstalk in the TME within these NSCLC models, independent of p53 status. We show that fibroblasts support tumor cells to modulate immune suppression within the TME *via* internalization of fibroblast-derived EVs. Furthermore, immune checkpoint blockade alters the hypoxic microenvironment and inhibits cell growth in the 3D-lung TME. Together, these data show that tumor-stromal crosstalk regulated by EVs and alterations within the TME can be monitored in 3D tumor models to better understand tumor biology and therapeutic response.

## Methods

### Cell Culture

A549 human lung adenocarcinoma cells were obtained from ATCC and cultured in DMEM (Dulbecco’s Modified Eagle’s Medium)/Hams F-12 50/50 (Corning, New York, NY) supplemented with 10% fetal bovine serum (FBS, Atlas Biologicals, Fort Collins, CO), 2 mM L-glutamine (1%, Corning), 100 IU/mL Penicillin/100 µg/mL Streptomycin (1%, P/S, MP Biomedicals, Solon, OH). H358 human lung bronchioalveolar carcinoma cells were purchased from American Type Culture Collection (ATCC, Manassas, VA) and cultured in RPMI 1640 media (Corning) supplemented with 10% FBS, 1% L-glutamine, and 1% P/S. IMR90 lung fibroblasts were obtained from Dr. Victor Thannickal and cultured DMEM (in 4.5 gm/L glucose) supplemented with 10% FBS, 1% L-glutamine, and P/S. Jurkat T cells were obtained from Dr. Olaf Kutsch and cultured in RPMI 1640 media (Corning) supplemented with 10% FBS, 1% L-glutamine, and 1% P/S. THP-1 human monocytes were purchased from ATCC and cultured in RPMI-1640 media modified with 10% FBS, 1% L-glutamine, penicillin/streptomycin (P/S) and 4.5 g/L of dextrose (Sigma-Aldrich, St. Louis, MO). THP-1 cells were then stimulated with 25 ng/mL of phorbol 12-myristate 13-acetate (PMA, Sigma-Aldrich) for 48 hours to transform them into non-committed M0 cells (21, 22). Baseline expression of HLA-DR (MHCII) and CD86 shown in **Supplemental Figure 3**. All cell lines were first validated to be mycoplasma negative using the PlasmoTest™ - Mycoplasma Detection Kit (Invivo-Gen, San Diego, CA). Cells were stained with the lipophilic dyes PKH26 and PKH67 (Sigma-Aldrich) following the manufacturer’s recommendations. Briefly, 1 mL diluent C was mixed with 1 μL of PKH dye (2 µM final concentration), and the cells diluted in 1 mL diluent C were added. This mixture was incubated at for 5 min at room temperature, in the dark. The labeling reaction was stopped by adding an equal volume (2 mL) of FBS. Cells were washed and pelleted for use.

### Generation of Extracellular Vesicle (EV) Depleted Media

RPMI 1640 or DMEM/Hams F-12 50/50media (Corning) was supplemented with 20% FBS and centrifuged on an ultracentrifuge overnight at 100,000 ×g at 4°C. Supernatant was filtered through a 0.2 µm cellulose acetate filter (Corning). The EV depleted media was then diluted 1:1 with base media to make a final concentration of 10% FBS and additional supplements were added to make complete THP-1 media (1% P/S, 1% L-Glutamine and (4.5 g/L of dextrose) or cell line specific media. EV depleted media was then stored at 4°C until use.

### Tumor Model Preparation and Bioreactor Setup

**Figure 1:**
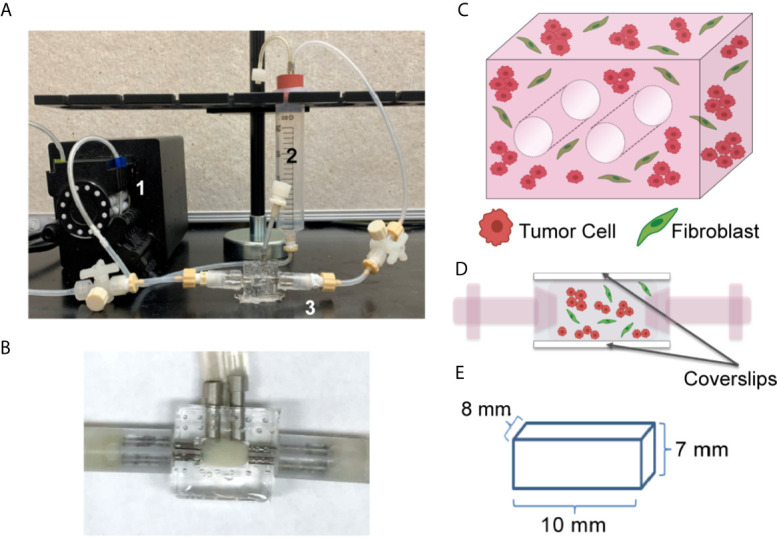
Tissue Engineered 3D Lung Cancer Models Established using a Perfusion Bioreactor Platform. **(A)** Circulating flow loop setup, showing peristaltic pump (1), media reservoir (2), and perfusion bioreactor (3). **(B)** Perfusion bioreactor containing ECM + cells within the middle chamber. **(C)** Schematic of the 3D volume, showing 2 through-channels, and mixture of tumor cells and fibroblasts (2:1 ratio). **(D)** Schematic of side view, showing cells within the central chamber of the bioreactor which has coverslip imaging windows on the top and bottom surfaces. **(E)** Dimensions of the ECM + cell 3D volume.

A549 or H358 and IMR90 cells (2:1 epithelial to fibroblast ratio, 5.25 × 10^5^ total cells/100 µl ECM) or tumor cells, IMR90 fibroblasts and THP-1 cells (1/3 THP-1+ 2/3 epithelial cells and fibroblasts (2:1 epithelial to fibroblast ratio), 5.25 × 10^5^ total cells/100 µl ECM) were mixed into an ECM containing 90% bovine collagen type I (Advanced Biomatrix, San Diego, CA) +10% basement membrane (growth factor reduced Matrigel (BM), Corning) - total protein approximately 6 mg/mL- and injected into a polydimethylsiloxane (PDMS, Sylgard 184, Krayden, Denver, CO) bioreactor (as shown in **Figure 1**). This volume was perforated by two 700 μm stainless-steel wires located within the upstream fitting. Following ECM polymerization, Teflon wires were removed, generating two, cannulated through-channels in the ECM/cell mixture. Surrogates were connected to a micro-peristaltic pump and a media reservoir *via* peroxide cured silicone tubing (Cole Parmer, Vernon Hills, IL), as previously described (23), and continuously perfused with 15 mL medium (645 µL/min for a shear stress of 1 dyne/cm^2^ per channel, tumor cell line specific or THP-1 EV depleted media) for 3 to 12 days (37°C, 5% CO_2_), with medium changed every 3 days. For treatment experiments, 10 µg/ml anti-PD-L1 blocking antibody or IgG control (BioXcell, Lebanon, NH) was included in the circulating media

### Isolation of Extracellular Vesicles From Circulating Media

EVs were isolated from bioreactor circulating media using the Total Exosome Isolation Reagent (for cell culture media, Invitrogen, Waltham, MA) per manufactures instructions. Briefly, circulating media was harvested and centrifuged at 300x g for 5 min to remove any circulating cells. Supernatant was collected and centrifuged at 2000 × g for 30 minutes to remove cells and debris. Supernatant was collected and 3 mL was mixed with 1.5 mL Total Exosome Isolation Reagent and incubated over night at 4°C. After incubation samples were centrifuged at 10,000 × g for 1 hour at 4°C to pellet EVs, the supernatant was discarded and the EV pellet was resuspended in 50 µL PBS.

### Analysis of Extracellular Vesicle Size and Concentration

The mean particle concentration and mean size of the EV were measured using a NanoSight NS300 (Malvern Panalytical, Westborough, MA). EVs were diluted 1:1000 with PBS and loaded onto a syringe pump for injection into the Nanosight. EVs were injected at pump speed of 50 at room temperature. 5 videos were acquired for each sample. All videos were acquired at a temperature of 23.2–23.3°C; camera level: 7; capture duration: 1 min/video; camera type: SCMOS; gain: 1; minimum tracks completed: 2000–4000/video; frames processed: 1951/video; frames per second: 25 fps; blur: auto; and detection threshold: 5.

### Evaluation of Hypoxia Using Hypoxisense-680 Staining and Imaging

The IVIS Lumina imaging system (Perkin Elmer, Waltham, MA) and Nikon A1R Confocal microscope were used to non-destructively image global and local fluorescence within 3D tumor models incubated with Hypoxisense 680 (Perkin Elmer) to monitor hypoxia. One day prior to imaging, 100 pM Hypoxisense 680 was injected into each 3D tumor model and incubated statically for 2 hours (37°C, 5% CO_2_). After dye incubation, media circulation was resumed. The following day 3D tumor models were sterilely disconnected from the flow loop and closed to surroundings in a laminar flow hood. 3D tumor models were imaged with the IVIS Lumina (Ex: 680/Em: 710, 1 second exposure, bin 8, f/stop 2.), and Nikon A1R (Objective: Plan Apo λ 10x, na 0.5, wd 4000; laser lines (nm): 405, 488, 567, 637) using Nis Elements 5.0 Imaging Software on days 5 and on day 10 before fixation, with Hoechst incubation prior to day 10 imaging and fixation. Identical square regions of interest (ROI) were drawn around tumor models to evaluate global fluorescent signal with the IVIS Lumina using Living Image Software (Perkin Elmer). Regional fluorescent signal was measured in confocal Z-stacks using the ImageJ open source processing package FIJI (24).

### ImageStream Flow Cytometry

Following growth for 7, 10 or 12 days, surrogates were removed from the bioreactor housing and a small portion was fixed for histologic analysis. The remaining 3D culture volume was mechanically digested for cell isolation. Briefly, cells were isolated from the 3D cultures by mincing the ECM containing cells and passing this mixture through a 40 micron cell strainer to isolate single cells. ImageStream combines flow cytometry with fluorescence imaging technology to resolve cells and EVs. The following antibodies were used to characterize EVs: PD-L1 PerCp-Cy5.5 (clone: MIH5, Invitrogen, Carlsbad, CA), EpCAM Alexa Fluor 594 (clone: 9C4, Biolegend, San Diego, CA), CD54 Fluor (clone: 24-31, eBioscience, San Diego, CA), CD81 PE-Cy7 (clone 5A6; BioLegend), CD63 eFlour450 (clone H5C6; Invitrogen). The following antibodies were used to characterize cells: CD64- PerCP-eFluor710 (clone: 10.1, Invitrogen), CD11b APC-Cy7 (clone: ICRF44, BD, Franklin Lakes, NJ), CD206-APC (clone: 15.2, BD), CD163 PE-Cy7 (clone: GH1/61, eBioscience). The stained EV samples were imaged at 60 × magnification with extended depth of field (EDF), while acquiring data on channels Ch01, CH02, Ch03, CH04, Ch06, Ch07, Ch09, Ch11 and Ch12. The stained cell samples were imaged at 60 × magnification with extended depth of field (EDF), while acquiring data on channels Ch01, Ch03, Ch06, Ch07, Ch09, Ch11 and Ch12. Appropriate controls, single color stains, and calibration beads were used to adjust spectral compensation. A total of 5000 events were acquired. Channels Ch01 and Ch09 were used as brightfields, and Ch12 or Ch6 was used for side-scatter. The acquired data was analyzed using IDEAS software version 6.2 (EMD Millipore, Bellerica, MA).

### Histologic Processing and Immunohistochemistry

Following growth for 7, 10 or 12 days, surrogates were fixed with neutral buffered formalin, processed to paraffin, and histological sections were prepared, as previously described (25). 5 micron sections were stained with hematoxylin and eosin (H&E) to evaluate cellular morphology and cell density (number of cells per cross-sectional area), and immunohistochemistry was performed to detect Ki-67 (1:100, clone Sp6, Thermo Scientific, Watham, MA), using the Dako Envision + Dual Link secondary detection kit (Agilent Technologies, Santa Clara, CA) containing the chromogen DAB, following antigen retrieval (10 mM citrate buffer, pH 6, Biogenex, San Ramon, CA).

### Image Analysis

Cell density, defined as the number of nucleated cells per 1 × 10^6^ pixels^2^ (area), was determined from photomicrographs (400x) of H&E-stained histologic sections of surrogates (minimum of 20 photomicrographs per sample, 3 samples per group). The number of nucleated cells was counted manually in FIJI and normalized to the cross-sectional area of each image, measured using the polygon tool in FIJI. A MATLAB script was developed to quantify signal intensity from imagestrips, allowing the cell of origin (the cell with the higher signal intensity) to be determined in double positive cell populations. This analysis was completed in a minimum of 100 cells per replicate.

### Statistical Analysis

The statistical analysis was performed using GraphPad Prism software (La Jolla, CA, USA). Data are presented as mean ± standard error of the mean unless indicated otherwise in the figure legend. A *p-*value less than 0.05 was considered to be statistically significant. A two-tailed unpaired Student’s *t*-test was used to evaluate statistical difference between two groups. One way ANOVA with Sidak’s multiple comparison testing was utilized to evaluate statistical difference for data with more than two groups.

## Results

We generated a lung tumor model that mimics the TME utilizing a bioreactor platform that replicates the 3D nature of the ECM and the interactions between diverse cell types within the TME. This platform allows for continuous perfusion of nutrients *via* a flow loop and peristaltic pump (**Figure 1A**), with the ECM-containing cells within the central chamber of the bioreactor (higher magnification image, **Figure 1B**). The culture chamber contains two 700 µm through-channels that function as pseudo-vessels for nutrient transportation (**Figure 1C**), and the bioreactor has side windows for noninvasive, real-time image analyses (**Figure 1D**). This system enables culture of a relatively large volume, with the culture chamber having a total volume of 560 mm^3^ (8 mm x 7 mm x 10 mm) (**Figure 1E**). The current platform includes the two cannulated microchannels within the culture volume and a larger surface area along the imaging window dimension to facilitate non-invasive imaging.

**Figure 2:**
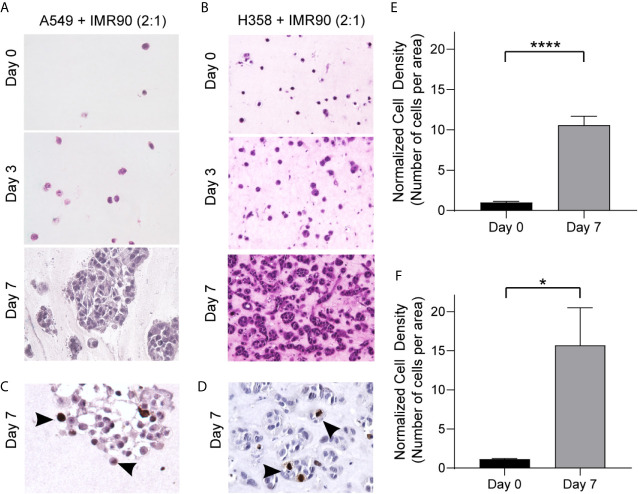
3D Culture Establishes a TME Allowing for Growth over 7 Days Culture. **(A, B)** Photomicrographs of H&E cross-sections showing increased cell density over time for tumor mimics containing, A549 tumor cells and IMR90 fibroblasts (2:1 ratio, **A**) or H358 tumor cells and IMR90 fibroblasts (2:1 ratio, **B**) within an ECM containing 90% enzyme extracted bovine collagen (~6 mg/mL) and 10% basement membrane proteins (growth factor reduced Matrigel). **(C, D)** Representative photomicrographs of immunohistochemical staining for Ki-67 following 7 days culture in A549 + IMR90 tumor mimics **(C)** and H358 + IMR90 tumor mimics **(D)** showing actively proliferating cells in both tumor mimics. **(E, F)** Cell density, or the number of nucleated cells per area, normalized to the starting (Day 0) cell density in A549 + IMR90 tumor mimics **(E)** and H358 + IMR90 tumor mimics **(F)** increases between days 0 and 7. n= 3-6 replicates per experiment. *p<0.05, **** p< 0.0001.

With this system, we generated two 3D models of lung adenocarcinoma, one using the A549 cell line, a KRAS mutant and p53 wild-type NSCLC cell line that resembles alveolar type II cells, and the other using the H358 cell line, which is a KRAS mutant, p53 null NSCLC cell line (26–29). Tumor cells alone in the 3D culture did not demonstrate optimal growth (**Supplemental Figure 1A**). To accurately model the lung TME and tumor cell-stromal crosstalk, we co-cultured lung cancer cells with IMR90 lung fibroblasts, at a 2:1 ratio of cancer cells to fibroblasts, within an ECM, containing collagen type I and basement membrane proteins (~6 mg/ml total protein). This combination of matrix proteins was chosen since collagens are abundant within the lung ECM contributing to the overarching architecture of the lung and aberrant collagen deposition is often found within lung cancers, yet basement membrane proteins are critical for epithelial cell function and dysregulated in cancer (30, 31). To optimize ECM composition, we first compared cell density, or the number of cells per area in histologic cross-sections, when cells were grown in a 9:1 or 1:1 ratio of collagen type 1 and growth factor-reduced Matrigel mixture. As shown in **Supplemental Figure 1B**, the 9:1 mixture was found to better support cell growth. Following model generation, cell growth was monitored over a 7 day culture period (**Figures 2A, B**) and immunohistochemical analyses of the proliferation marker Ki-67 showed proliferating cells in both models following 7 days culture (**Figures 2C, D**). Additionally, cell density was found to increase over time with both models having a significantly increased cell density on histologic cross-sections at day 7 compared to day 0 (co-culture initiation, **Figures 2E, F**).

**Figure 3:**
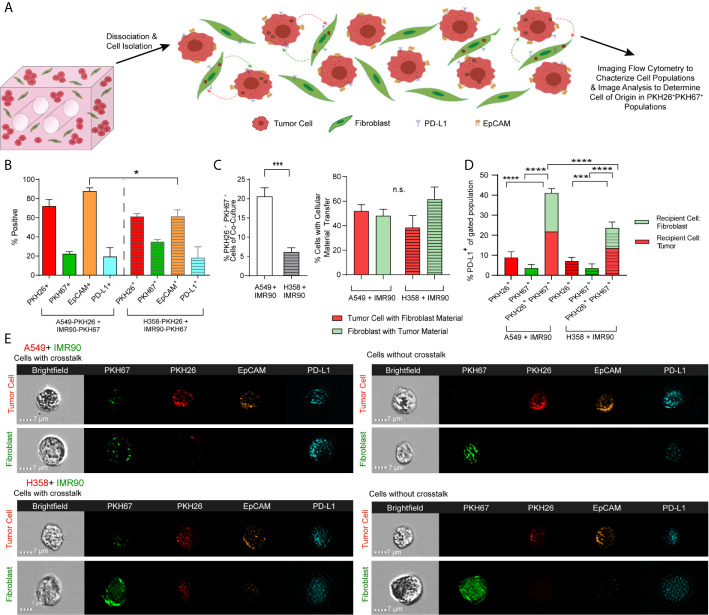
Tumor-Stromal Crosstalk Occurs within Lung Cancer Mimics. **(A)** Cartoon representation detailing cell dissociation following co-culture and subsequent imaging flow cytometry to characterize cell populations and image analysis to determine the cell of origin in PKH26^+^PKH67^+^populations. **(B)** Characterization of models of both p53 null and p53 wild-type lung carcinoma shows variability in cell populations between models. **(C)** Increased tumor-stromal crosstalk, *via* internalization of cell material, is seen in the p53 wild-type model (left), but the percentage of tumor cells containing fibroblast derived EVs versus the percentage of fibroblasts that contain tumor cell derived EVs, or the double positive cell populations is similar (right). **(D)** PD-L1 expression is increased in cell populations with cell crosstalk *via* material transfer (PKH26^+^PKH67^+^) when compared to cell populations without cell crosstalk (PKH26^+^ or PKH67^+^). **(E)** Representative Imagestrips showing cells with cell crosstalk, with transfer of fibroblast derived material (as shown by punctate PKH67 signal) to tumor cells (PKH26 labeled) or tumor cell derived material (as shown by punctate PKH26 signal) to fibroblasts (PKH67 labeled, left) compared to cells without crosstalk (right). n= 3 replicate bioreactors run in triplicate per experiment. ns, not significant, *p<0.05, ***p<0.001, ****p< 0.0001.

To characterize cell-cell communication in the lung TME, tumor cells and fibroblasts were pre-labeled with lipophilic dyes, PKH26 (red) and PKH67 (green), respectively, co-cultured for 12 days and analyzed using imaging flow cytometry (ImageStream) to characterize cell populations and determine the extent of material transfer between tumor cells and fibroblasts (illustrated in **Figure 3A**). When markers of each cell subset were analyzed, tumor marker, EpCAM, was found to be higher in the p53 wild-type (A549) model when compared to the p53 null (H358) model, suggesting alteration in the proportion of tumor cells and fibroblasts and outgrowth of tumor cells within this model (**Figure 3B**). We assessed expression of immunomodulatory protein, programmed death ligand 1 (PD-L1), as it confers immune tolerance through interaction with immune checkpoint protein programmed death 1 (PD-1) on infiltrating T cells within the TME (32). The proportion of cells expressing PD-L1 was similar in both tumor models (**Figure 3B**), suggesting that the immunomodulatory potential is similar in these lung cancer models. Interestingly, baseline expression of PD-L1 in A549 was significantly lower than the level observed in H358 and fibroblasts (**Supplemental Figure 2**), yet the % PD-L1^+^ cells were similar after co-culture indicating modulation of the immunoregulatory processes *via* tumor-fibroblast interactions. We then assessed whether tumor-fibroblast interactions elicit cell-cell transfer based on the PKH26 and PKH67 signals marking the transfer of labeled cellular material from one cell population to the other (*i.e.* from fibroblasts to tumor cells and vice versa). The A549 model demonstrated an increase in PKH26^+^PKH67^+^ cells (**Figure 3C**, left). Image analysis was then utilized to determine which cell type was the recipient cell and to better define the observed tumor-fibroblast crosstalk. This analysis showed that both tumor cells and fibroblasts function as recipients of exchanged cellular material in both lung cancer cell models, with tumor cells functioning as the cell of origin more frequently in the H358 model, as shown by the increased proportion of fibroblasts containing tumor cell material (**Figure 3C**, right). When PD-L1 expression was evaluated within tumor cells or fibroblasts with or without material transfer or crosstalk (as indicated by PKH dyes), an increase in the percentage of PD-L1^+^ cells was observed in the PKH26^+^PKH67^+^ cell population in both models, compared to cells without material transfer (PKH26^+^PKH67^-^ or PKH26^-^PKH67^+^, **Figure 3D**); the proportion of PKH26^+^PKH67^+^PD-L1^+^ cells in the A549 model was significantly higher compared to the H358 model. We further evaluated if one cell type within the PKH26^+^PKH67^+^PD-L1^+^ cells was driving this increased expression of PD-L1, and found that within this cell population both fibroblasts and tumor cells were equally likely to be the recipient cell (**Figure 3D**). This is further observed in representative image strips, which show punctate regions of PKH67 signal within tumor cells that were pre-stained with PKH26 and EpCAM^+^, as well as punctate regions of PKH26 and EpCAM within some fibroblasts that were pre-stained with PKH67 (**Figure 3E**)

**Figure 4:**
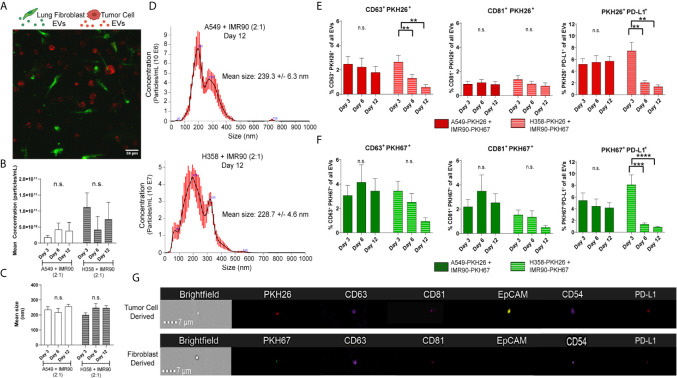
Extracellular Vesicles within Circulating Media have Immunomodulatory Potential. **(A)** Confocal image from day 3 of culture, showing PKH26 labeled tumor cells (red) and PKH67 labeled fibroblasts (green) in co-culture within the bioreactor. **(B, C)** No changes in mean concentration **(B)** or size **(C)** were found in either tumor model with EV particle analysis. **(D)** Representative particle analyses for each tumor model. **(E)** Tumor cell-derived EVs (PKH26^+^) express common exosome markers (CD63 and CD81) as well as PD-L1. **(F)** Fibroblast-derived EVs (PKH67^+^) express common exosome markers (CD63 and CD81) as well as PD-L1. **(G)** Representative imagestrips of tumor cell-derived and fibroblast-derived EVs showing PKH signal as well as expression of common EV markers and PD-L1. n=3-9 replicates per time point. ns, not significant, **p<0.01, *** p<0.001, ****p<0.0001.

Next, we determined whether EVs mediated the material transfer between tumor cells and fibroblasts and if this cellular crosstalk partially contributed to the modulation of PD-L1 observed in the engineered TME. As p53 has been shown to regulate EV production, we hypothesized that the p53 status of the cancer cell lines utilized in our tumor models may influence EV-mediated tumor-stromal crosstalk. We and others have previously reported that the H358 cell line produces fewer EVs when compared to p53 wild-type cell lines (17, 33), yet how p53-mediated EV production may be regulated in co-culture with stromal cells is not known. Therefore, we evaluated EV mediated cell-cell communication in our model using the culture parameters described above. Confocal microscopy showed cellular location, with tumor cells and fibroblasts distributed throughout the 3D matrix (**Figure 4A**). EVs were then isolated from circulating media and mean EV concentration (**Figure 4B**) and size (**Figure 4C**) within each model were determined using NanoSight particle analysis (representative size/concentration plots, **Figure 4D**). While the mean size remained similar between models, fewer EVs were present in the A549 model. EVs were then evaluated by ImageStream flow cytometry to characterize tumor cell- and fibroblast-derived vesicle populations. The presence of common exosome markers, tetraspanins CD81 and CD63 which are present on a fraction of exosomes and can be used to differentiate exosomes from other EVs (34), as well as PD-L1 were evaluated in PKH26^+^ (red, tumor) or PKH67^+^ (green, fibroblast) EVs from each model isolated following 3, 6, and 12 days culture (**Figures 4E, F** **;** representative image strips, **Figure 4G**). CD81^+^ and CD63^+^ EVs derived from both tumor cells and fibroblasts were found within both models, suggesting EV release and potential EV internalization by both cell types. Additionally, both tumor cell- and fibroblast-derived EVs were PD-L1^+^, suggesting immunomodulatory potential of these EVs. Interestingly, differences in the dynamics of EV secretion were observed between models, with both tumor cell- and fibroblast-derived PD-L1^+^ EVs being high at day 3 but declining at days 6 and 12 within the p53 null (H358) model compared to the p53 wild-type model (A549), which showed stable levels of derived PD-L1^+^ EVs throughout culture.

**Figure 5:**
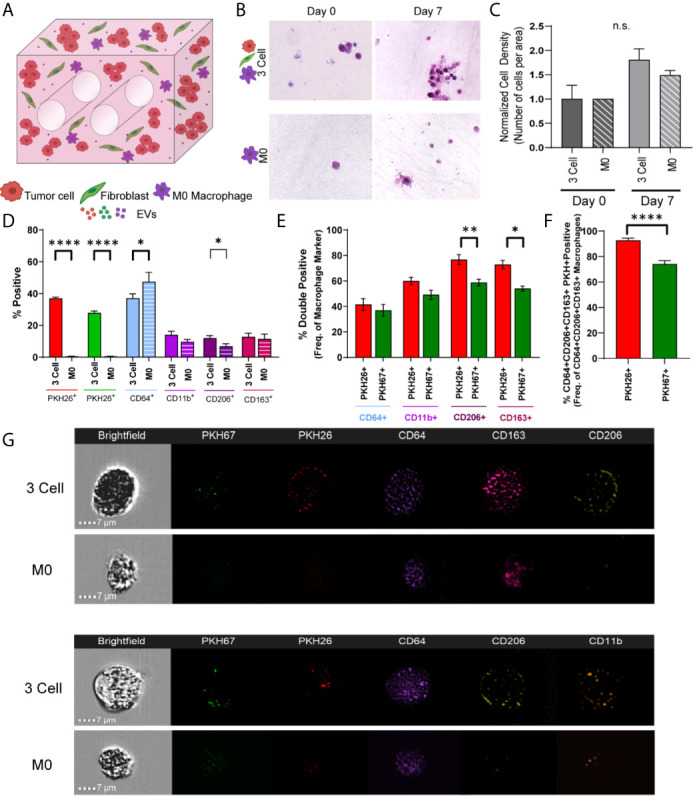
Tumor Cells and Stromal Fibroblasts Polarize M0 macrophages within an Immune Cell Reconstituted Model of Lung Carcinoma. **(A)** Schematic showing immune cell reconstituted model (3 Cell) containing tumor cells (red), fibroblasts (green), and M0 macrophages (PMA activated THP-1, purple). **(B)** Representative photomicrographs showing cell density within 3 cell tumor mimics (top) over time, compared to models containing only M0 macrophages (bottom). **(C)** Quantification of cell density, normalized to day 0. **(D)** Characterization of cell populations within 3 cell and bioreactors containing M0 macrophages showing increased CD206^+^ cells within 3 Cell bioreactors compared to bioreactors containing M0 macrophages only following 7 days culture. **(E)** Characterization of M0 macrophages containing tumor derived material (indicated by PKH26 signal) or fibroblast derived material (indicated by PKH67 signal) showing a significant increase in the PKH26^+^CD206^+^ and CD163^+^ cell populations. **(F)** M2 like macrophages (CD64^+^CD163^+^CD206^+^) contain more tumor cell-derived material (indicated by PKH26 signal). n=3-6 replicates per condition. **(G)** Representative imagestrips showing PKH signal within M0 macrophages. ns, not significant, *p<0.05, **p<0.01, ****p<0.0001.

As immune cells within the TME are known to modulate tumor progression and the cell and EV data indicate cellular crosstalk and immunomodulatory potential within these lung tumor models, we next generated immune cell reconstituted models of lung adenocarcinoma by adding M0 macrophages (PMA activated THP-1) to our tumor-fibroblast models (**Figure 5A**). Again, tumor cells and fibroblasts were pre-labeled with PKH26 (red) and PKH67 (green), respectively, and M0 macrophages were added so that 1/3 of the cells incorporated were immune cells. First, cell density on histologic cross-sections was evaluated and a trend towards increased cell density was observed from day 0 to day 7 within the bioreactors containing all 3 cell types (H358 + IMR90 + M0) compared to bioreactors with M0 only (**Figures 5B, C**). We then determined macrophage polarization *via* ImageStream flow cytometry following 7-day culture. As M2-like tumor associated macrophages (TAMs) are commonly found in the TME of lung carcinoma, M2 markers CD206 and CD163 were evaluated (35, 36). A significant increase in CD206^+^ cells was found within bioreactors that contained all 3 cell types compared to those with M0 only (M2 macrophage markers, CD64, CD11b, CD206 and CD163, **Figure 5D**). Further, when M0 cells were evaluated for tumor cell- or fibroblast-derived material (indicated by PKH signal), tumor cell-derived PKH26 was found to be significantly increased in M0 cells expressing CD163 and CD206 when compared to fibroblast-derived PKH67, suggesting tumor cells skew M0 cells to polarize towards M2-like macrophages (**Figure 5E**). Furthermore, when evaluating PKH signal with pro-tumor M2 like macrophage markers, tumor cell-derived PKH26 was found within a higher proportion of M2 like macrophages when compared to fibroblast-derived PKH67 (**Figure 5F**); this is further demonstrated by ImageStream flow cytometry, where punctate PKH26^+^ regions are observed within CD64^+^ macrophages but few PKH67^+^ regions are observed (representative image-stream strips, **Figure 5G**). Similar results were observed when the A549 cell line was utilized in co-culture with lung fibroblasts and M0 macrophages (**Supplemental Figure 4**).

**Figure 6:**
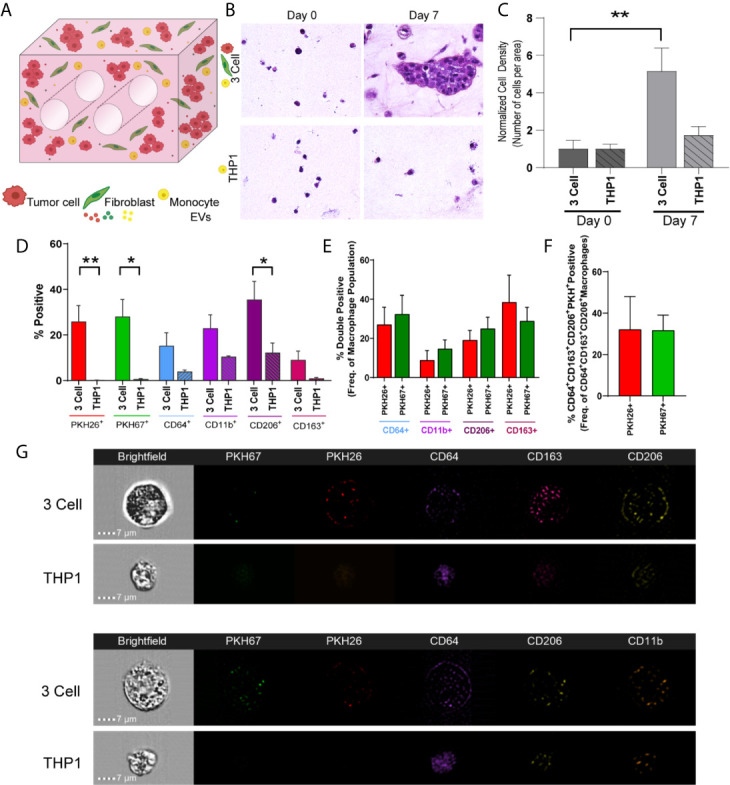
Tumor Cells and Stromal Fibroblasts Polarize Monocytes within an Immune Cell Reconstituted Model of Lung Carcinoma. **(A)** Schematic showing immune cell reconstituted model (3 Cell) containing tumor cells (red), fibroblasts (green), and monocytes (THP-1, yellow). **(B)** Representative photomicrographs showing increased cell density within 3 cell tumor mimics (top) over time, compared to models containing only THP-1 cells (bottom). **(C)** Quantification of cell density, normalized to day 0, indicating cell growth within 3 cell tumor mimics. **(D)** Characterization of cell populations within 3 cell and THP-1 bioreactors showing increased CD206+ cells within 3 Cell bioreactors compared to THP-1 bioreactor following 7 days culture. **(E)** Characterization of monocytes containing tumor derived material (indicated by PKH26 signal) or fibroblast-derived material (indicated by PKH 67 signal). **(F)** No significant difference is observed between PKH26^+^ (tumor cell-derived material transfer) and PKH67^+^ (fibroblast-derived material transfer) M2 like macrophages (CD64^+^CD163^+^CD206^+^). n=3-9 replicates per condition. **(G)** Representative imagestrips showing PKH signal within THP-1 monocytes. *p<0.05, **p<0.01.

When undifferentiated THP-1 monocytes were added to the engineered TME, increases in cell density and CD206^+^ cells were observed from day 0 to day 7 (**Figures 6A–C**; M2 macrophage markers, CD64, CD11b, CD206 and CD163, **Figure 6D**). Further, when THP-1 monocytes were evaluated for tumor cell- or fibroblast-derived material (indicated by PKH signal), tumor cell-derived PKH26 and fibroblast derived PKH67 were found at similar levels within THP-1 cells expressing CD206 and CD163, suggesting tumor cells and fibroblasts educate monocytes to differentiate and polarize towards an M2 phenotype (**Figure 6E**). Similarly, both PKH26 and PKH67 were found within monocytes that expressed CD64, CD206, and CD163 (**Figure 6F**). This is supported by the observation of punctate PKH26^+^ and PKH67^+^ regions within undifferentiated CD64^+^ THP-1 monocytes following co-culture with tumor cells and fibroblasts (representative image strips, **Figure 6G**). While no significant changes in CD206 or CD163 expression were observed when the A549 cell line was co-cultured with lung fibroblasts and THP-1 monocytes, tumor cell-derived PKH26 and fibroblast-derived PKH67 were within THP-1 cells expressing CD163 and CD206, suggesting tumor and fibroblast education of monocytes in this model as well (**Supplemental Figure 5**). Interestingly, tumor cell-derived PKH26 was found within a higher proportion within these cell populations, suggesting that the tumor cells may be more responsible for skewing monocytes to polarize towards M2-like macrophages within this model.

**Figure 7:**
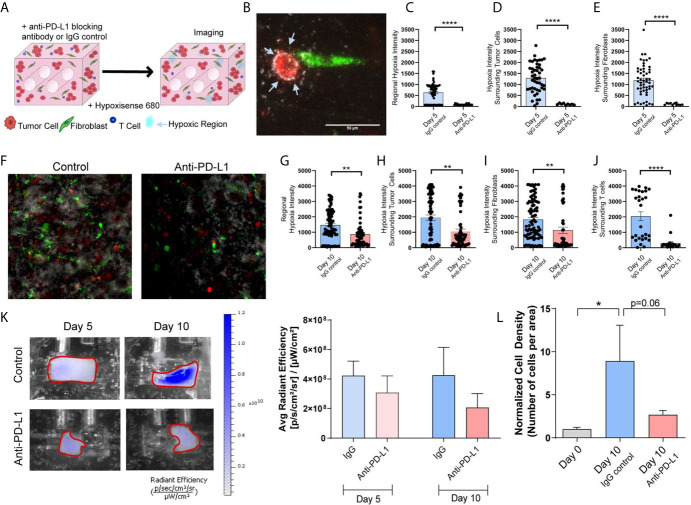
Immune Checkpoint Blockade Impacts Regional Hypoxia within Tumor Mimics **(A)** Schematic showing tumor mimics containing tumor cells (red), fibroblasts (green) and T cells (blue) which are treated with anti-PD-L1 blocking antibody or IgG control and undergo hypoxia analysis using a probe for carbonic anhydrase IX (Hypoxisense 680). **(B)** Representative confocal image showing tumor cell (red) and fibroblast (green) with carbonic anhydrase IX positive region (white) around the tumor cell. **(C)** Measurement of regional hypoxia within confocal images shows a decrease in the average intensity of hypoxic regions, indicating decreased hypoxia with anti-PD-L1 treatment on day 5 of culture. n=23-62 regions per condition. **(D, E)** Measurement of hypoxic regions surrounding cells within confocal images shows a decrease hypoxia surrounding tumor cells **(D)** and fibroblasts **(E)** with anti-PD-L1 treatment on day 5 of culture. n=9-52 regions per condition. **(F)** Representative confocal image showing decreased carbonic anhydrase IX signal within the anti-PD-L1 treated tumor mimic following 10 days culture. **(G)** Measurement of regional hypoxia within confocal images shows a decreased hypoxia with anti-PD-L1 treatment on day 10 of culture. n=58-74 regions per condition. **(H–J)** Measurement of hypoxic regions surrounding cells within confocal images shows a decrease hypoxia surrounding tumor cells **(H)**, fibroblasts **(I)**, and T cells **(J)** with anti-PD-L1 treatment on day 5 of culture. n=20-86 regions per condition. **(K)** Left: IVIS imaging of carbonic anhydrase IX probe, Hypoxisense 680, on days 5 and 10 of culture indicates a decrease in global hypoxia within anti-PD-L1 treated tumor mimics. Right: Quantification of the signal from IVIS imaging shows a trend towards decreased global hypoxia within anti-PD-L1 treated tumor mimics. n=7-9 replicates per condition. **(L)** Cell density, normalized to day 0, shows a decrease in cell growth with anti-PD-L1 treatment. n=3-7 replicates per condition. *p<0.05, **p<0.01, ****p<0.0001.

Next, we evaluated whether tumor-stromal crosstalk in our model would replicate immunosuppression and T cell exhaustion encountered in the TME, and if immune checkpoint blockade (ICB) in this model modulates biochemical cues elicited by tumor-stromal crosstalk that promote immunosuppression. The impact of ICB on tumor growth/progression was determined by co-culturing Jurkat T cells with H358 NSCLC cells and IMR90 lung fibroblasts. Tumor cells and fibroblasts were pre-labeled with PKH26 (red) and PKH67 (green), respectively, and Jurkat T cells were added so that 1/3 of the cells initially incorporated were T cells. The anti-PD-L1 blocking antibody (10 µg/ml) or IgG control were added to the circulating media following model setup (**Figure 7A**). To monitor hypoxia, the hypoxia dye HypoxiSense 860 (Perkin Elmer) which recognizes carbonic anhydrase IX, a protein commonly upregulated in hypoxic regions, was injected into the culture volume 1 day prior to imaging. Non-invasive imaging was performed with the In Vivo Imaging System (IVIS) Lumina platform and confocal microscopy was used to monitor global and regional hypoxia signals, respectively, on days 5 and 10. Confocal images showed regional hypoxic areas (depicted in white) localized around tumor cells (red) within the engineered TME (**Figure 7B**). Quantitation of the average intensity of hypoxia at day-5 showed a marked reduction in regional hypoxia in response to PD-L1 blockade (**Figure 7C**); similar reductions in hypoxic intensity were seen around tumor cells (**Figure 7D**) and fibroblasts (**Figure 7E**) following anti-PD-L1 treatment. This effect on tissue hypoxia was sustained, with similar results obtained on day-10 of culture (**Figures 7F–J**). Hoechst was added to the culture volume for the final imaging time point to stain the nuclei and better visualize cellular location, as PKH signals dilute out with cell proliferation; this enabled the evaluation of hypoxia surrounding T cells at this later time-point. Significant decreases in regional hypoxia (**Figure 7G**), as well as hypoxia surrounding tumor cells (**Figure 7H**), fibroblasts (**Figure 7I**), and T cells (**Figure 7J**) were observed. These results were confirmed with IVIS imaging, where global hypoxia also trended towards a decrease with the addition of anti-PD-L1 when compared to the IgG control (**Figure 7K**). Additionally, histologic cell density was evaluated to determine the impact of checkpoint blockade on cell growth. While the IgG control treated samples showed a significant increase in cell density compared to the starting cell density (day 0), no significant increase was observed with anti-PD-L1 treatment (**Figure 7L**), validating the tumor-suppressive effect of checkpoint blockade therapy and modulation of cellular crosstalk that results in mitigation of tissue hypoxia in our engineered tumor model.

## Discussion

Tumor cell behavior is determined by the tissue microenvironment and cell-cell interactions, yet replicating this in *ex-vivo* cell culture systems remains a major challenge. This may account for the disappointing success in clinical translation of candidate interventions that have largely relied on traditional 2D culture models with a single cancer cell lineage (37). Multicomponent 3D tumor models better mimic the complex tumor-microenvironment interactions. We generated a model system that allows evaluation of tumor-fibroblast-immune cell crosstalk; this engineered microenvironment closely mimics the lung TME by including tumor-associated dimensionality, multiple cell populations, and the ECM. These components, along with the capacity to modulate the mechanical and biochemical microenvironments, better approximate the TME *in vivo* allowing for the study of complex cellular interactions. Our models build upon previous models of breast carcinoma (23, 38), and differ from existing 3D lung cancer models that include immune cells (39), in that we are able to evaluate direct tumor-stromal crosstalk and not simply response to conventional interventions within static culture. In generating perfused NSCLC lung tumor models that differ in p53 status, we are able to differentiate tumor-stromal crosstalk and EV-mediated cell crosstalk between these models. Our observations of material transfer, by both tumor cells and fibroblasts suggests EV internalization by cells in the engineered TME and highlights differences between the two NSCLCs, with greater internalization of EVs in the A549 model compared to the H358 model.

PD-L1 expression by tumor cells is a well-established mechanism of immune suppression within lung tumors (40). Fibroblasts within tumors, or CAFs, also express PD-L1 and this stromal population, as well as physical and chemical cues from the TME can modulate PD-L1 expression by tumor cells (41–45). PD-L1 expression is known to vary by cell line, which we confirm here with baseline expression of PD-L1 being substantially lower in the A549 cell line (46). Yet, following co-culture PD-L1 levels are similar between models, suggesting immunomodulatory potential is influenced by cross-talk between tumor cells and fibroblasts within our models. While tumor derived EVs can suppress the immune system *via* PD-L1 both within the local TME and systemically, the potential for PD-L1^+^ CAF-derived EVs to modulate the immune system is unknown (47). Fibroblast-derived EVs can express PD-L1 and package PD-L1 into EVs in pathological states (48, 49). The increase in PD-L1^+^ tumor and fibroblast EVs in our model shows that the tumor-fibroblast crosstalk is involved in the regulation of immune suppression within the lung TME.

While tumor-infiltrating immune cells within the TME affect lung cancer outcome, the factors that influence the composition and function of the immune landscape within the TME remain poorly defined. We evaluated the influence of EV-mediated tumor-stromal crosstalk on monocytes and show that both tumor cell-derived and fibroblast-derived EVs polarize monocytes towards an M2-like phenotype to generate a tumor promoting microenvironment. Therefore, both tumor cells and CAFs not only directly influence response to therapy, but also indirectly shape the immune landscape by educating monocytes. These phenotypic changes can modulate expression of PD-L1 within the immune cell compartment, including macrophages, which in turn, could influence the efficiency of ICB (50).

ICB therapies targeting programmed cell death-1 (PD-1, commonly expressed on T cells within the TME) or its ligand (PD-L1, commonly expressed on tumor and stromal cells within the TME), have been shown to rescue effector CD8^+^ T cell function for effective tumor cell killing (51). While the TME is known to influence response to immunotherapy (52), the impact of immune-targeted therapies on biochemical aspects of the TME, including hypoxia, nutrient availability and cytokine signaling, has not been well characterized. These factors may alter T cell recruitment and retention of T cell subsets within the TME that ultimately impact tumor growth and response to therapeutic intervention. Hypoxia within the TME suppresses anti-tumor immunity; oxygen deficiency negatively impacts the energetic demands needed for optimal immune responses and induces mitochondrial defects that augment T cell exhaustion within the TME (53). While there is evidence for hypoxia-dependent regulation of PD-L1 and consequent response to checkpoint blockade, the impact of anti-PD-L1 therapy on the hypoxic microenvironment has not been determined (54, 55). With our tumor models, we defined the impact of ICB on hypoxia within the TME. Reductions in regional and cell specific carbonic anhydrase IX levels, and cell density in response to ICB, indicate that this therapy not only blocks direct immune modulation, but also reduces the hypoxic phenotype of cells within the treatment area. The mechanism regulating this process is unknown; the decreased tumor mass alone may relieve the oxygen depletion, or independent factors such as ECM alterations in response to ICB may be occurring, as ECM remodeling has been linked to immune changes in the TME (56). Additionally, the extent of hypoxia has been shown to be influenced by the tolerance of individual cells to hypoxia and EVs released by distant cells can modulate this tolerance (57). Therefore, we could speculate that cells responding to ICB may become tolerant to hypoxia and release EVs that decrease hypoxia in surrounding cells, however further studies are necessary. Recapitulation of the hypoxic response within our 3D tumor models, similar to *in vivo* solid tumors, provides a unique advantage of this *ex vivo* culture system without the need for subjecting cultures to hypoxic stress, commonly adopted in 2D cultures.

The 3D tumor models described recapitulate key aspects of the lung tumor TME, while reducing complexity by not including all stromal cell subtypes. This allowed for specific tumor-fibroblast and tumor-immune interactions to be defined, without the overlapping crosstalk between other cell subsets. While this does not fully mimic the autologous nature of the TME in a human tumor, it allows for more precise delineation of tumor-stromal interactions. The allogeneic interactions within these cell line models do not fully recapitulate tumor-immune interactions expected within the TME. The ICB therapy used within this study successfully targeted the PD-L1 expressing tumor cells and fibroblasts, therefore the allogeneic nature of this model likely has minimal impact on the tumor-fibroblast interactions. Additionally, while the models generated differ in p53 status, differences in other tumor suppressor pathways, including STK11/LKB1, are known to impact immune regulation within the TME and should be further studied in systems like the one described here (58). Moving forward, additional studies evaluating specific EV cargo-mediated regulation of macrophage/monocyte polarization and hypoxia by ICB are warranted. Our 3D bioengineered models may be used, not only to elucidate novel mechanisms of tumor-stromal crosstalk, but to determine the pre-clinical efficacy of novel therapeutics with greater predictive success.

## Data Availability Statement

The original contributions presented in the study are included in the article/**Supplementary Material**. Further inquiries can be directed to the corresponding author.

## Author Contributions

KG was involved in bioreactor and experimental design and execution, data collection, data analysis, and manuscript and figure preparation. HA was involved in experimental execution, data collection, data analysis, and manuscript editing. AW was involved in data collection, data analysis, and manuscript editing. YW was involved in data collection and manuscript editing. KH was involved in data collection, data analysis, and manuscript editing. SB developed the MATLAB script utilized for data analysis and was involved in manuscript editing. MA was involved in experimental design and manuscript editing. JB was involved in bioreactor design and production and manuscript editing. SP was involved in experimental design and manuscript editing. VT provided reagents and was involved in experimental design and reviewing the overall concepts and thought process of the manuscript. JD was involved in experimental design and oversight, data interpretation, and manuscript and figure preparation. All authors contributed to the article and approved the submitted version.

## Funding

Research reported in this publication was supported by the National Cancer Institute Cancer Center Support Grant P30 CA013148 and used the UAB High Resolution Imaging Facility, the UAB Comprehensive Flow Cytometry Core Facility (NIH P30 AR048311 and NIH P30 AI27667), the UAB Small Animal Imaging Facility (NIH P30 CA013148 and 1S10OD021697), and the UAB Pathology Core Research Lab. This project was supported by 5T32HL105346-10 (KG), BCRFA awarded to JD and JB, and HL114470-06 award to VT.

## Conflict of Interest

The authors declare that the research was conducted in the absence of any commercial or financial relationships that could be construed as a potential conflict of interest.
