# Supplementary material for: Extracellular Vesicle Mediated Tumor-Stromal Crosstalk Within an Engineered Lung Cancer Model
Source: Front Oncol. 2021 Apr 23;11:654922. doi: 10.3389/fonc.2021.654922 (PMC8103208; doi:10.3389/fonc.2021.654922)
Supplement: Supplementary file 1 [file DataSheet_1.pdf]

Supplemental Figures and Figure Legends

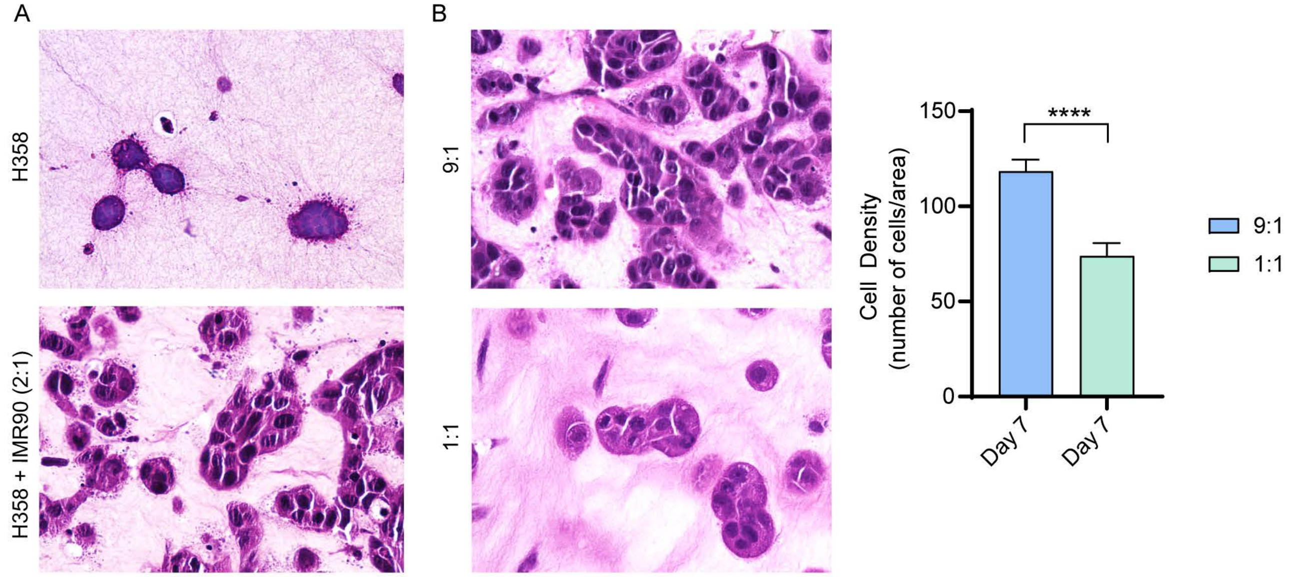

Supplemental Figure 1: Addtion of Lung Fibroblasts and Optimization of ECM **A**. H&E stained cross-sections (400x) showing H358 cells culture alone (top) or with IMR90 lung fibroblasts (bottom, 2:1 ratio of tumor cells to fibroblasts) within a 3D ECM showing increased growth and maintence of appropriate architechture with the addtion of fibroblasts following 7 days culture. **B**. Left: H&E stained cross-sections (400x) showing co-culture of tumor cells and fibroblasts (H358 + IMR90) within a 9:1 (top) or 1:1 (bottom) mixture of collagen type 1 and GFR Matrigel (basement membrane) following 7 days culture. Right: Cell density within histologic sections shows increased cell growth following 7 days culture with cells culture in the 9:1 mixture compared to the 1:1 matrix mixture. n= minimum of 20 ROI evaluated. **** p<0.001.

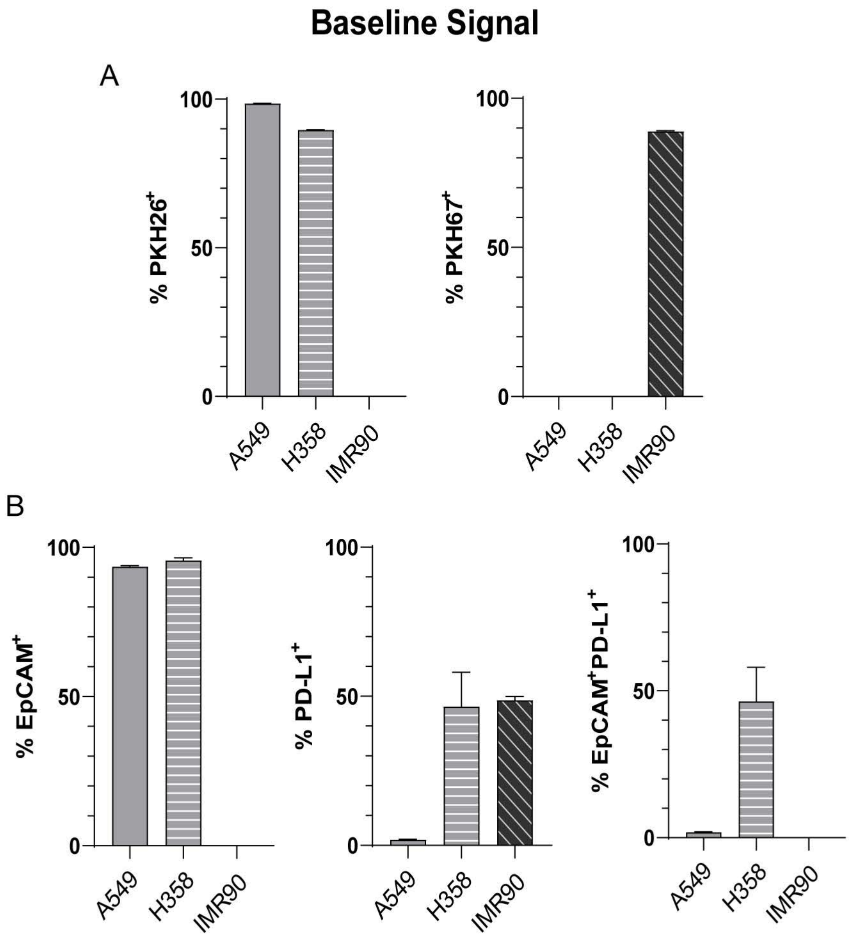

Supplemental Figure 2: Baseline signal before co-culture. **A**. Time 0 signal of PKH26 (left) and PKH67 (right) within cell populations. **B**. Time 0 expression of EpCAM (left), PD-L1 (middle), and EpCAM and PD-L1 (right) within cell populations.

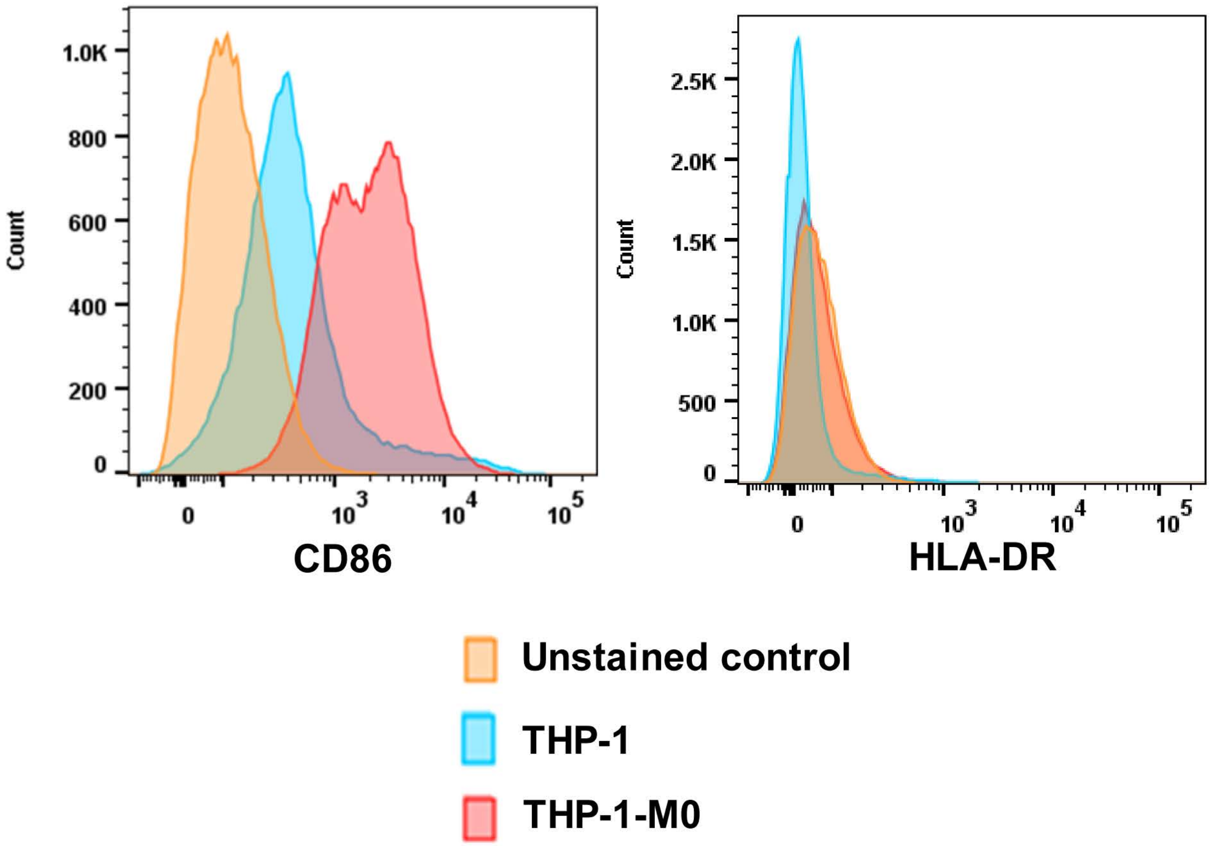

Supplemental Figure 3: Expression of CD83 and HLA-DR (MHCII) on THP-1 cells and PMA activated THP-1 cells (THP-1-M0).

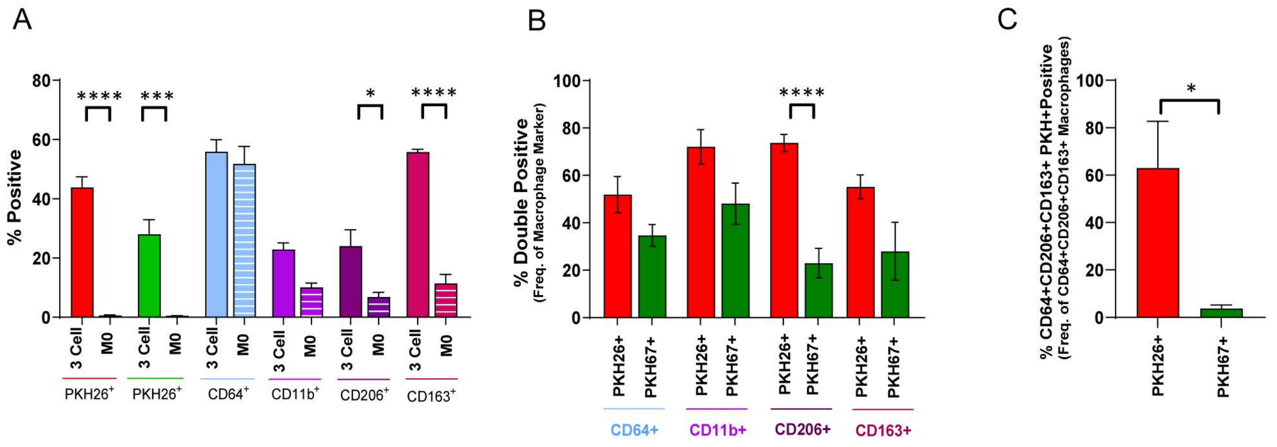

Supplemental Figure 4: A549 Tumor Cells and Stromal Fibroblasts Polarize M0 macrophages within an Immune Cell Reconstituted Model of Lung Carcinoma. **A**. Characterization of cell populations within 3 cell (A549-PKH26 + IMR90-PKH67 + PMA-Activated THP-1(M0)) and PMA-Activated THP-1 bioreactors. **B**. Characterization of M0 macrophages containing tumor derived (indicated by PKH26 signal) and fibroblast derived (indicated by PKH67 signal) material, showing a significant increase in the CD206^{+} cell containing tumor cell derived material. **C**. M2 like macrophages contain more tumor cell-derived material (indicated by PKH26 signal). n=3-6 replicates per condition. *$p<0.05$, *** $p<0.005$, **** $p<0.0001$.

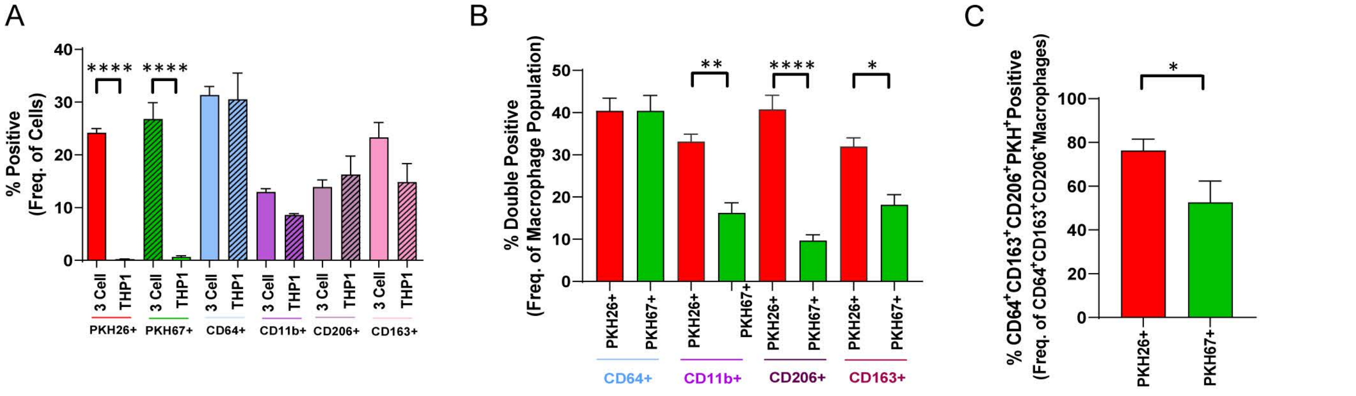

Supplemental Figure 5: A549 Tumor Cells and Stromal Fibroblasts Polarize Monocytes within an Immune Cell Reconstituted Model of Lung Carcinoma. **A**. Characterization of cell populations within 3 cell (A549-PKH26 + IMR90-PKH67 + THP-1) and THP-1 bioreactors. **B**. Characterization of monocytes containing tumor derived (indicated by PKH26 signal) and fibroblast derived (indicated by PKH67 signal) material. **C**. M2 like macrophages contain more tumor cell-derived material (indicated by PKH26 signal). n=3-6 replicates per condition. *p<0.05, **** p<0.0001.
